# A qualitative inquiry into the challenges and complexities of research supervision: viewpoints of postgraduate students and faculty members

**Published:** 2015-07

**Authors:** ALIREZA YOUSEFI, LEILA BAZRAFKAN, NIKOO YAMANI

**Affiliations:** 1 Medical Education Department, Medical Education Research Center, Isfahan University of Medical Sciences, Isfahan, Iran;; 2 Medical Education Research Center, Isfahan University of Medical Sciences, Isfahan, Iran

**Keywords:** Research supervision, Medical students, Grounded theory, Qualitative

## Abstract

**Introduction:**

The supervision of academic theses at the Universities of Medical Sciences is one of the most important issues with several challenges. The aim of the present study is to discover the nature of problems and challenges of thesis supervision in Iranian universities of medical sciences.

**Methods:**

The study was conducted with a qualitative method using conventional content analysis approach. Nineteen faculty members, using purposive sampling, and 11 postgraduate medical sciences students (Ph.D students and residents) were selected on the basis of theoretical sampling. The data were gathered through semi-structured interviews and field observations in Shiraz and Isfahan universities of medical sciences from September 2012 to December 2014. The qualitative content analysis was used with a conventional approach to analyze the data.

**Results:**

While experiencing the nature of research supervision process, faculties and the students faced some complexities and challenges in the research supervision process. The obtained codes were categorized under 4 themes Based on the characteristics; included “contextual problem”, “role ambiguity in thesis supervision”, “poor reflection in supervision” and “ethical problems”.

**Conclusion:**

The result of this study revealed that there is a need for more attention to planning and defining the supervisory, and research supervision. Also, improvement of the quality of supervisor and students relationship must be considered behind the research context improvement in research supervisory area.

## Introduction

The academic thesis is actually the most symbolic example of research project by students (1, 2). While doing research, the students express their knowledge, skills, attitude, power, initiative, confidence and perseverance along with their own research spirit in their academic thesis (3, 4). Therefore, the academic thesis can be considered as the first systematic empirical step of research, introduced to the students (5, 6). One of the most effective ways of creating a learning relationship between students and tutors is the process of thesis supervision by the supervisor. It also establishes a clear objective for training and clarifies the role of the students and supervisors (7, 8). In the thesis supervision process, there are a number of important and complex elements which empowers the tutor for effective supervision (9). These ways include creating a professional relationship, urging the students to study, helping them in research topic selection and preliminary design research, assisting them in their personal and general problems, and last but not the least helping them in implementing and completing the survey (9, 10).

Another important aspect of research supervision which has been investigated is the quality of guidance given by the guiding professor (11-14). The studies have shown that various factors, such as theoretical and practical knowledge of conducting a research and the communication skills used in teacher-student relationship can affect the quality of the thesis supervision and the editing process. Like any other multi-factorial concept, in addition to these factors, there are also a number of underlying unknown factors which can alter the quality and results of the teacher supervision and guidance (15). Safaee Movahed et al. (2010) explored supervisors’ norms involving the selection of supervisors by graduate students in a qualitative and phenomenological research by semi-structured interviews for data collection. Supervisors’ norms consisted of supervisor selection which was classified into six categories: behavior, academic position, non-academic features, credible support, and limitations. Results demonstrated that students acquired these norms through performance of supervisors’ observation in teaching and dissertation committees as well as informal networks such as senior students (14).

Changiz et al. (2003) also studied the obstacles the students were engaged in when doing research and thesis activities. In their study, the views of 131 faculty members who had experienced the editing and implementation process of 298 academic theses were gathered. This study showed that approximately over 50% of tutors didn’t assign enough time to reviewing and correcting the thesis. According to this study, over 40% of the faculty members considered financial problems, administrative difficulties for proposal approval and lack of technical supports, such as statistical consultations, the main obstacles in the way of research process while over 50% believed that students’ lack of time, attention and inconsistency of decisions made by different levels of supervising committees were the barriers to research (16). After reviewing the literature on student-supervisor relations in the years 1990 to 2009, Evans and Stevenson (2010) found out that the quality of such relationship experiences is mainly influenced by two factors: the level of clarity in expectations and the support given to students' supervisors. They suggest that teachers' guidance activities at micro- and macro- levels guide the students through the hard and mysterious pathways of research so that each thesis project can create a personal vision (17). In conclusion, literature reviews show that the students` supervision and guidance in thesis process, which is a very important issue in higher education, faces many complex challenges. The aim of this study is to discover the nature of these problems and challenges in Iranian universities of medical sciences. The real question in hand is that how the teachers and students experience the problems and dilemmas in this process. The purpose of this study is to identify themes that explain thesis supervision complexities and challenges through the postgraduate medical sciences students and faculties in Iran.

## Methods

The study was conducted with a qualitative method using content analysis approach. The design is appropriate for this study because it allows participants to describe their experiences centered on factors that may improve the quality of thesis supervision in their own words. For data gathering, semi-structured interviews were administered. Key informants in purposeful sampling consisting of 30 people including 19 faculty members of basic and clinical sciences and 11 postgraduate students (Ph.D students and residents) were selected according to a variety of academic ranks, work experiences and specialty degrees. Sampling started with purposive sampling method with maximum variation (e.g. variation in genders, discipline, and academic ranks) and continued with theoretical sampling. The data were collected using semi-structured in-depth interviews. 

Interviews began with general topics, such as "talk about your experiences in research supervision" and then the participants were asked to describe their perceptions of their expertise. Probing questions were also used to deeply explore conditions, processes, and other factors that participants recognized as significant. The interview process was largely dependent on the questions that arose in the interaction between the interviewer and interviewees. In the process of the study, after obtaining permission from the participants, the interview was recorded and transcribed verbatim immediately. The interviews were conducted in a private and quiet place and in a suitable time when the participants felt comfortable. Then, verification of documents and coordination for subsequent interviews were done. The participants’ demographic data were also recorded. The interviews lasted for one hour on average and each interview was conducted in one session with the interviewer's notes or memos and field notes. Another method of data collection in this study was an unstructured observation in the educational setting.  The investigator observed the way of interactions among faculty members and students. 

The interviews were conducted from September 2012 to December 2014. Each participant was interviewed for one or two sessions. The mean duration of the interviews was 50 minutes. To analyze the data, we used MAXQDA software (version 10, package series) for indexing and charting. Also, we used qualitative content analysis with a conventional approach to analyze the data. In content analysis at the first, semantic units should be specified, and then the related codes should be extracted and categorized based on the similarity. Finally, in the case of having a high degree of abstraction, the themes can be determined. Content analysis method is used to verify the existence of certain words and concepts in the text for giving structure and discipline to the data. In conventional approach, use of predetermined classes is avoided and classes and their names are allowed to directly come out of the data. To do so, we read the manuscripts and listened to the recorded data for several times until an overall sense was attained. Then the manuscript was read word by word and the codes were extracted. At the same time, the interviews were continued with other participants and coding of texts was continued and sub codes were categorized within the general topics. Then the codes were classified in categories based on their similarity (18, 19). The categories which were similar were classified in more general categories and each category was given a name. To ensure the accuracy of the data, we used peer review, check member, the researchers' acceptability, and the long and continuing evaluation through in-depth, prolonged, and repeated interviews, using the colleagues’ comments. The number of initial codes from the coding stage in the process of data analysis was reduced to 354 codes. These codes, based on the conceptual similarities and differences, were summarized and classified into 3 main categories and subcategories in the axial coding stage. The major themes included "Contextual problem", "Role ambiguity", "Poor reflection in supervision" and “Ethical problem" (Table 1).

To improve the accuracy and rigor of the findings, Lincoln and Guba’s criteria, including credibility, dependability, confirm ability, and transferability were used (20). The researchers tried to increase the credibility of the data by keeping prolonged engagement in the process of data collection and analysis, collecting data from two major referral centers for patients who had suicide attempts, writing memos, confirming the accuracy of data analysis by 3 specialists in the field of qualitative research and checking original codes by some participants to compare the findings with the participants’ experiences. To increase the dependability and confirmability of data, maximum variation was observed in the sampling. Also, to increase the power of data transferability, adequate description of the data was provided in the study for critical review of findings by other researchers.

**Ethical considerations**

The present study was approved by the Ethics Committee of Shiraz University of Medical Sciences. The aim of the research and interview method was explained to the participants and the informed consent for interview and its recording was obtained. In all stages of present study, Data maintaining was done in order to keep participants confidentiality. 

## Results

The average age of faculty members in this study was 42.34±14.60 years and all of them were married and the average age of students in this study was 29.54±2.60 years and all of them were married. The main categories of data included the Contextual problems, Role ambiguity, Poor reflection and Ethical problems.

**Table 1:** Themes and sub-themes used in the interview

**Themes**	**sub-themes**
**Contextual problem**	The workload of supervisor
	Poor staff developments
	Lack of resource
**Role ambiguity **	Week structure of thesis supervision
	Ambiguity in expertise criteria in supervision
**poor reflection **	Ineffective evaluation
	Lack of self-assessment
	Lack of reflection on and in action
**Ethical challenge**	Inefficient communication
	Lack of professional behaviors
	Formation of negative interactions
	Incompetent students

**Contextual problems**

Understanding the contextual problems in thesis supervision was one of the important results mentioned by the participants. The subthemes emerged in this category includes factors related to workload of supervisor, lack of resource, and the poor staff developments.

**The workload of supervisors**

The workload of supervisors was the other challenge for supervisor and supervisee. Due to the lack of time, thesis supervisors and advisors have not enough time to guide and counsel students and carry out their duties. This is due to the students’ overload in universities and the improper proportion of students and supervisors. 

One of the faculty members stated: *“…for being an expert supervisor, we needs time, and we do not have enough time for supervision and learning its necessary skills ...”* (Faculty member No.5).

**Lack of resource**

The other problem which was emphasized by both faculty members and students was the financial support of students at universities, and inadequate administrative procedures for limited access to information resources. The students who write the thesis pay much, spend a lot of time, but do not receive enough support. One participant who had experienced many problems in an experimental research said:

*“…Because I had to use a device which didn’t exist in Iran and you know universities don’t pay any budget for these expenses." Of course, it is not always like this, and in some cases students have to change their topic which takes a long time...”* (Student No.5).

**Poor staff development**

The participants in this study repeatedly confirmed that there was a need to design supervisor development programs which are acceptable and have the standards for the changing research contexts. The supervisors’ knowledge, methods and techniques can also affect the quality of the dissertation. Unfortunately, the knowledge in young faculty members isn't enough for supervising the students, especially for medical residents. 

*“…The training courses are not useful and purposeful, nor do they result in enhancing practical ability. …, some courses must be designed based on individual needs, and perhaps in order to be an experienced and expert advisor, there is a need for an internship period.”* (Faculty member No.3).

Contextual problems were the main concern of students and faculties in research supervision. Most of the participants had experienced insufficient scientific/library and human resources in their thesis writing. So the stakeholders must be able to manage and control the mentioned problems. Control and supervising these issues lead to effective supervising in thesis supervision and prevent students from wasting time and energy which can result in their disappointment and publication of low quality thesis. 

**Role ambiguity**

The participants in this study stated that the improper choice of supervisor, due to weak structure of thesis supervision, irregular meetings of professors and students, and lack of attention to thesis, poor and unstructured guidance, lack of systematic and insufficient guidance, and insignificant allocation of time to supervision were the major problems in this area.

**Weak structure of thesis supervision**

"The most important problems include lack of format for advice and guidance, lack of attention to the existing format, lack of specific planning for professors and students’ duties, and lack of planning for professors and students interaction" (Faculty member No.1).

Also, the participants focused on a structured set of tasks that the supervisor and students must do.  One of the participants said: *“…What do students expect from me? What should I do for the students? What should the students do for me*?*.. ..”* (Faculty member No.5).

**Ambiguity in expertise criteria in supervision**

Some faculty members and students stated that evidence of the competence or incompetence of professors and the measure of this capability are not clear enough. Expertise is derived from three essential elements of knowledge, skill, experience, and the ability to solve problems in thesis project. However, measuring and understanding the capabilities is not possible with the existing standards.

In this respect, one of the faculty members stated:

*“…It is not right to evaluate an instructor’s level of skill and knowledge merely based on   the number of articles he or she publishes. Having more published articles doesn’t necessarily mean being a better teacher. In other words, being a good teacher is more about how these articles were written rather than the number of them. Unfortunately, the quality does not matter most of the time...”* (Student No.3).

According to the participants’ experiences in the area of research activities, academic staff and medical students have serious weaknesses in defining the good problems, choosing the appropriate method for research, data analysis, interpretation of results, and writing scientific papers and there is not coherent and compiled program to enhance research capabilities. Determining of academic staff and students’ role in thesis activities can lead to enhancement of individuals in their competency in research.

**Poor Reflection**

This category emerges from the following subcategories: "Ineffective evaluation", "Lack of reflection on and in action", "Inefficient communication" and "Week self-assessment in faculty members and students".  

**Ineffective evaluation**

One of the major problems of our research supervision at the University is non-standard evaluation criteria, lack of expertise in thesis judgment and scoring, and inadequate standards in supervision and evaluation of faculty members in this area.

*“…Almost there was no monitoring on my work; I myself chose a topic, worked on it by error and trial, and after a lot of suffering I presented my thesis...”* (Student No.4).

**Lack of self-assessment**

The participants in this study often pointed out the way self-assessment can improve the research abilities in the supervisor and the supervisee, while some of them have not enough self-assessment. *“…Only teachers themselves can know and improve their own weaknesses. No other third party is able to determine the abilities and qualifications of a supervisor. I think, in any thesis process which includes writing, supervision and guidance, a self assessment must be done both by the students and teachers...”* (Faculty member No.14).

**Lack of reflection on and in action**

Based on the participants’ views, supervisors and students agree on the role of reflection in thesis writing, and almost all of them stated that they had a big gap in desirable and current condition in this area.  

*“…A supervisor mustn’t have a tunnel vision because tunnel vision leads to linear vision and we do not see all dimensions of a problem; in supervisory processes, we need reflection and in-depth thinking ...”* (Faculty member No.2).

*“…This is the most important problem to us. A lot of students have concluded that they have special mental frames towards special subjects. The most important barrier to the mind is the supervisor him/herself. Some advisors pass their opinions day by day and they prefer to reach new victories every day. These advisors are preferred by students and are a golden chance...”* (Student No.10).

The results of this study demonstrated that participants agreed that they did not have enough experience, self-assessment and reflection which are major factors in higher education. Reflection in both students and instructors can lead to solve the probable problems which may occur while project is being conducted.

**Ethical problems**

This sub-theme reflects the inefficient communication, lack of professional behaviors, formation of negative interactions and incompetent students.

**Inefficient communication**

Lack of enough communication, either verbal or by email, was among influential factors effecting supervising process. Based on the participants’ views, factors such as listening to students and considering their comments, listening to supervisors, and considering their correcting comments need enough reflection. 

As one of the student put it *“…I don’t mean that he or she is a bad person; what I mean is that even a knowledgeable and skillful supervisor can’t succeed unless he or she can interact with others; otherwise, it is clear that they can’t even defend their own work, let alone their students’. (Or they can’t even take what is rightfully theirs, let alone defend their students...”* (Student No.1).

**Lack of professional behaviors**

 Lack of professional behaviors was an important issue in this study on which faculty members and students agreed. Unprofessional behaviors occurred in every stage of thesis supervision, in supervising, in the beginning of the project or end of it. One of the faculty members stated that: "In class, I always pay attention to ethics and it is valuable for university and science" (Faculty member No.2).

Or another faculty mentioned: *“…A student may learn something but not ethics which is very dangerous...”* (Faculty member No.5).

The Students emphasized the misuse of students by supervisor in some areas; for example, *“…*The reason that supervisors accept a thesis is to use students; students are like robots and writing articles is the only goal of writing thesis*...”* (Student No.7).

**Formation of negative interactions**

Students expressed their dissatisfaction with the supervision and complained *“…It was a bad feeling, a very bad feeling, frustration and depression. When thesis is traded and no one understands the bias, my job future is the same as this, too...”* (Student N0.5).

*“…The advisor doesn’t know anything and he/she is miserable. If an advisor has ethical commitment in his/her job, he/she will never give a work to students which they can’t do it and face a lot of problems due to it. In my opinion, ethical commitment is so important...”* (Student No.4).

**Incompetent students**

Some of the students don’t consider studying just as a duty and because of financial problems they have to do some jobs. Also, incompetent students refer to those who have difficulty in communication, knowledge, and the required skill for doing the task.

*“…Sometimes feeling interferes with our task as supervisors, when I see the problem of student in managing life and research and thesis writing, communication...”* (Faculty member No.12).

Based on participants’ experiences, ineffective communication and absence of a positive pattern and professional behavior can lead to the formation of an improper behavior in students and the outcome would be incompetent students in thesis writing. In order to solve this dilemma, it would be necessary to pay attention to different ethical aspects in research supervision process. 

## Discussion

This study revealed some complexities and challenges in research supervision in Iranian medical sciences universities. The major themes included "unstructured supervision and role ambiguity in thesis supervision", "contextual and ethical problems" and "poor reflection in supervision" in research supervision.

For the first category, weak structure of thesis supervision emerges from the workload of supervisor, ambiguity in expertise criteria in supervision, lack of enough research method knowledge, and ineffective thesis evaluation subcategory. 

So, based on the participants’ views, research supervision challenges in our universities are more related to systemic dilemmas; these problems are particularly about university professors as supervisors or advisors, non-structured and non-experts, students’ research projects without any structure and supportive planning. This result of our study is in line with those of other studies, such as that conducted by Changiz, et al. (16).

Wiscer (2005) in a constructive manner determined the duty of supervisors by three stages of the supervising process and stated the duties of supervisor in each of them. According to him, at the starting stage of guidance process, proposals would be prepared. After that supervision maintaining would be done and finally activities such preparing papers, building self steem and self confidence in students to answer possible questions and making them ready to enter higher levels of thinking would be done (15).

The Second category was contextual and ethical problems emerging from lack of resource, poor staff developments, lack of professional behaviors, absence of good models, formation of negative interactions, and incompetent students. Based on the results of this study, lack of resources, non-professional behaviors and negative students-professors, professors-professors and students-students interactions can lead to ambiguity of roles and unclear tasks for the supervisor and supervisee. Several studies point to the fact that lack of resources and ambiguity of roles can lead to non-professional behaviors (21, 22).

In a recent study, the improper environment contextual problem, which produces research climate with negative interpersonal interactions in some situations, such as faculty members and students interaction, peer interaction and unethical behavior, has been mentioned (23-25).

Good communication in research supervision is the key element of supervisory task. In this regard, Baltzersen et al. (2014) in a recent empirical study found that discussions between the student and supervisor about the supervision process have a positive impact on the quality of the communication.  These perspectives are then used to discuss what specific types of meta-communication might facilitate good supervision in higher education. It is suggested that one should distinguish between meta-communication as a part of transparent communication style and meta-communication about the collaboration period in supervision (12). Also, Lee, in his research, states that supervisors of doctorate students are trying to resolve the tensions between their professional role as an academic and their personal self as well as to encourage the students to move along a path towards increasing independence. The concepts are examined in the light of each of these tensions. Finally, the research sheds light on the power of the supervisor’s own experience as a student; it is suggested that supervisors need to be aware of both positive and negative aspects of each of these conceptual approaches (9).

According to the participants, in this research, lack of time and inaccessibility of the supervisor, due to having multiple academic and non-academic duties in multiple activities, such as teaching, were the other problems. In a another  study in Iran, it was found that supervisors work in public and private clinics in addition to their academic activities; thus, limited time is left for supervising and guiding the students.

The participants of this study recommended that we build a critical and reflective community of postgraduate supervisory who can develop their supervision practice through reflective conversations. In a study by Vilkinas (2008), twenty-five faculty members were interviewed to determine how they supervised their Ph.D. students’ thesis preparation. A content analysis of the interview data indicated that the majority of them were task-focused. They supported their students intellectually, emotionally, and structurally. Some academics considered their students as colleagues, and a few developed research teams. Watching the students grow, develop and do research with them as colleagues was the most enjoyable aspects of the supervision process (13).

The participants wished to create a professional development opportunity that could enhance the supervisors’ capacity to manage the ongoing interpersonal and academic complexity of the supervision process, as well as its dynamic character. 

Lack of formative evaluation and feedback was also mentioned; this was consistent with another study in Iran and in other countries on this issue (26-30). In Kathryn’s research, it was found how conflicting knowledge of cultures and values negotiated in supervisory practices influenced the processes and outcomes of the supervisory rela­tionships (30). So, as the participants mentioned, problems related to ambiguity of roles and lack of reflection in students and supervisors can make several challenges in research supervision process.

The limitation in this research was limited access to students from other universities in the country.  Another limitation was difficulty and dilemma for some students to state their actual problems, due to some considerations with regard to their universities. 

## Conclusion

 Problems and challenges in research supervision process are caused by improper and unstructured context and the educational climate in which the tasks and responsibilities of individuals are not clear and well defined. This situation can lead individuals to indifference and lack of critical thinking and reflection. Doing a thesis requires a suitable context of all aspects and responsibility and responsiveness of both students and supervisors. Actually, guidance and supervision of theses has lost its logical process and thesis supervision is done only based on a hidden curriculum. 

## Footnotes

Authors’ Contributions: Leila Bazrafkan developed the study design, conducted the interviews and analysis, ensured trustworthiness, and drafted the manuscript. Alireza Yousefi as the supervisor participated in the study design, supervised the codes and data analysis process, and revised the manuscripts. Nikoo Yamani as the research consultant participated in the study and advised during the study.
